# First results of hormone receptors' status in Malagasy women with invasive breast cancer

**DOI:** 10.11604/pamj.2014.17.153.3045

**Published:** 2014-03-03

**Authors:** Nomeharisoa Rodrigue Emile Hasiniatsy, Clairette Raharisolo Vololonantenaina, Hary Fanambinantsoa Rabarikoto, Narindra Razafimanjato, Hasina Dina Ranoharison, Joelson Lovaniaina Rakotoson, Luc Hervé Samison, Florine Rafaramino

**Affiliations:** 1 Service Oncologie-Hématologie-Radiothérapie HUJRA, Antananarivo, Madagascar; 2 Service Anatomie Pathologique Institut Pasteur de Madagascar, Antananarivo, Madagascar; 3 Service Gynécologie Obstétrique CENHOSOA, Antananarivo, Madagascar; 4 Service de Chirurgie Thoracique HUJRA, Antananarivo, Madagascar; 5 Service d'Imagerie Médicale HUJRA, Antananarivo, Madagascar; 6 Service de Médecine Interne, HUJRB, Antananarivo, Madagascar; 7 Service Chirurgie Viscérale HUJRA, Antananarivo, Madagascar

**Keywords:** Breast cancer, hormone receptors, Institut Pasteur de Madagascar, Malagasy, status

## Abstract

**Introduction:**

Breast cancer is the most common malignancy tumor amongst Malagasy women registered at the pathology unit of the “Institut Pasteur de Madagascar”. In Madagascar, there is no laboratory practicing hormone receptors' status on these tumors. Until now no study about hormone receptors' status of Malagasy women with invasive breast cancer was performed. So it will be the first study talking about this topic. The aim of this study was to determine hormone receptors' status in Malagasy women with invasive breast cancer.

**Methods:**

This retrospective and descriptive study was based on patients' medical files from 2009 to 2011. It included all invasive breast cancer diagnosed in Malagasy women at the pathology laboratory located at the “Institut Pasteur de Madagascar”, in Antananarivo. Along this period this laboratory has sent paraffin blocks of invasive breast carcinoma in two pathological laboratories in France.

**Results:**

We collected 77 cases of invasive breast cancer along this period. The mean age was 48.8 +/- 10.7, ranging from 26 years to 70 years. There were 46.8 % (n = 36) women with progesterone receptor positive (PR+), 53.2 % (n = 41) with progesterone receptor negative (PR-). For the estrogen receptor, 61.0 % (n = 47) were positive and 36.4 % (n = 28) were negative. ER+/PR+ represented 44.2 % (n = 34); ER-/PR- 33.8 % (n = 26); ER +/ PR- 16.8 % (n = 13); ER-/PR+ and ER-/PR- represented respectively 2.6 % (n = 2).

**Conclusion:**

Patients in our study had more important rate of ER-, PR- and a less important rate of ER+/PR+, PR+. These results suggest that more study related to Hormone Receptor profile should be conducted in Malagasy women with breast cancer.

## Introduction

Breast cancer ranks first amongst cancers seen at the “Institut Pasteur de Madagascar” (IPM), either for women or for all locations combined [1]. In Madagascar there is no laboratory practicing hormone receptors' status in malignant breast tumors. Despite this lack of hormone receptors status determination, hormonotherapy is prescribed for these patients; and according to Rafaramino et al. they represented 41.69 % of women with breast cancer in her study [2]. From 2009 until now, the pathological laboratory of the IPM regularly sent in two French laboratories, located in Paris, paraffin blocks of invasive breast cancer to determine hormone receptors status. Up to now, no study about hormone receptors' status of breast cancer in Malagasy women with invasive breast cancer was performed. So this will be the first study talking about this topic. The aim of this study was to determine hormone receptors' status of Malagasy women with invasive breast cancer.

## Methods

**Data collection**: Patients were recruited according to the following steps:

Patients registered in the cancer registry of the IPM from 2009 to 2011; Blocks were sent and analyzed in France; After Hematoxylin Erythrosine Safran study, block used for the immunohistochemical study were selected, it has been practiced ABC peroxidase, DAB revelation with Automate Ventana Benchmark in presence of the following antibodies: E-cadherin; Ki-67; Estrogen Receptor (ER); Progesteron Receptor (PR); Oncoprotein cerB2.

Patients with immunohistochemistry results of at least one HR were retained; Patients with PR or ER staining more than 1 % were considered positive (PR+; ER+). The results were registered on EPI6-INFO version 3.5.3 of the Center Diseases Control and Prevention, Atlanta before analysis.

**Inclusion criteria**: Breast tumors diagnosed in IPM during the study period (date of receipt of biopsy specimens, surgical specimens or blocks to be analyzed at the IPM); Axillary lymph nodes sampling or other specimens with diagnosis of malignant invasive breast cancer; Blocks sent to IPM by other pathological laboratory for immunohistochemistry study.

**Non inclusion criteria:** Blocks sent to France after December 31st, 2011; Axillary lymph nodes sample and tumors which were analyzed without malignant histology; Non-invasive breast tumors; Biopsy of permeation nodules or any other samples outside the mammary gland.

**Exclusion criteria:** Patients whose specimens have been revised twice (first time for a biopsy and a second time for a mastectomy). They were taken into account for an exam; blocks sent to the IPM without pathology report or clinical information; Specimen in which the result of immunohistochemistry studies were not contributory; cases for which data were unavailable.

## Results

We have collected 77 files of patients in our study during 3 years. The mean age was 48.8 years +/-10.7, ranging from 26 years to 70 years with a median age of 47 years. The mean age was respectively for patients with ER-, ER+, and ER- 47.7 years old, 50.7 and 47.5 without statistic differences (p-value = 0.1). For the PR status, mean age was 46.8 years for PR- and 51.1 years for PR+; there was no statistic differences (p-value = 0.07). The most frequent histological type was the ductal invasive carcinoma with 87.0 % (n= 67) (Table 1).

**Table 1:** Age and histological type of 77 women with invasive breast cancer at the “Institut Pasteur de Madagascar” 2009 to 2011

Items	Characteristics	Number	Percentage ( %)
Age at diagnosis (years)	< 35	7	9.2
	35 – 50	36	46.7
	> 50	34	44.1
	Total	77	100.0
Histological type	Invasive ductal carcinoma	67	87.0
	Invasive lobular carcinoma	5	6.5
	Other	5	6.5
	Total	77	100.0

**For all population**: There were 46.8 % (n = 36) women with PR+, 53.2 % (n = 41) with PR-. For the ER, 61.0 % (n = 47) were ER+ and 36.4 % (n = 28) were ER-. We were unable to have ER status for two women. ER+/PR+ represented 44.2 % (n = 34); ER-/PR- 33.8 % (n = 26); ER+/PR- 16.8 % (n = 13); ER-/PR+ and ER-/PR- represented respectively 2.6 % (n = 2) of this population (Table 2, Table 3).

**Table 2:** The estrogen receptor and progesterone status of 77 women with invasive breast cancer seen at IPM since 2009 to 2011, for all population and according to age category

Age group	Descriptive variable		Number	Percentage ( %)
All population	ER	ER+	47	61.0
		ER-	28	36.4
		ER?	2	2.6
		TOTAL	77	100.0
	PR	PR+	36	46.8
		PR-	41	53.2
		TOTAL	77	100.0
< 35 years	ER	ER+	1	14.3
		ER-	6	85.7
		TOTAL	7	100.0
	PR	PR+	1	14.3
		PR-	6	85.7
		TOTAL	7	100.0
35-50 years	ER	ER+	22	61.1
		ER-	13	36.1
		ER?	1	2.8
		TOTAL	36	100.0
	PR	PR+	17	47.2
		PR-	19	52.8
		TOTAL	36	100.0
> 50 years	ER	ER+	24	70.6
		ER-	9	26.5
		ER?	1	2.9
		TOTAL	34	100.0
	PR	PR+	18	52.9
		PR-	16	47.1
		TOTAL	34	100.0

**Table 3:** Distribution of the 77 women with invasive breast cancer from 2009 to 2011 in IPM depending on the hormone receptor status, for all population and according to age group

Age group	Hormone receptor status	Number	Percentage ( %)
All population	ER+/PR+	34	44.2
	ER-/PR-	26	33.8
	ER+/PR-	13	16.8
	ER-/PR+	2	2.6
	ER?/PR-	2	2.6
	Total	77	100.0
< 35 years	ER+/PR+	1	14.3
	ER-/PR-	6	85.7
	ER+/PR-	0	0.0
	ER-/PR+	0	0.0
	ER?/PR-	0	0.0
	Total	7	100.0
35 – 50 years	ER+/PR+	16	44.4
	ER-/PR-	12	33.3
	ER+/PR-	6	16.7
	ER-/PR+	1	2.8
	ER?/PR-	1	2.8
	Total	36	100.0
> 50 years	ER+/PR+	17	50.0
	ER-/PR-	8	23.5
	ER+/PR-	7	20.6
	ER-/PR+	1	2.9
	ER?/PR-	1	3.0
	Total	34	100.0

**For patients < 35 years old**: From 7 patients included (9.2 %); one of them was ER+ and PR+; the others (n = 6) were ER- and PR-. The HR status of the first patient was ER+/PR+ and the others were all ER-/PR-.

**For patients 35-50 years old**: They represented 46.7 % (n = 36) of our population. There were 47.2 % (n = 17) women with PR+, 52.8 % (n = 19) with PR-. For the ER, 61.1 % (n = 22) were ER+ and 36.1 % (n = 13) were ER-. We were unable to have ER status for one patient for this category of age. ER+/PR+ represented 44.4 % (n= 16); ER-/PR- 33.3 % (n = 12); ER+/PR- 16.7 % (n = 6); ER-/PR+ and ER-/PR– represented respectively 2.8 % and 2,8 % (n = 1).

**For patients > 50 years old**: This age group represented 44.1 % (n = 34) of our population. There were 52.9 % (n = 18) women with PR+, 47.1 % (n= 16) with PR-. For the ER, 70.6 % (n = 24) were ER+ and 26.5 % (n = 9) were ER-. We were unable to have ER status for one patient. ER+/PR+ represented 50.0 % (n= 17); ER-/PR- 23.5 % (n = 8); ER+/PR- 20.6 % (n = 7); ER-/PR+ and ER-/PR- represented respectively 2.9 % (n = 1) and 3.0 % (n = 1) of this age group.

**Analysis:** Statistical analysis was performed between age and positivity of HR, but it was not possible because of insufficient patient number.

## Discussion

Our study suggests that Malagasy women with breast cancer have more important rate of ER-, PR-; and less important rate of ER+/PR+ and PR+. This retrospective study we have conducted is the first document analyzing the status of Malagasy women with invasive breast cancer towards HR. The first explanation is that until now,, there officially is no laboratory in the Malagasy territory which practices HRS in this type of tumor while breast cancer ranks first in terms of frequency of cancers diagnosed at the IPM [1]. This population cannot be considered as representative of the Malagasy population, because the patients have to pay the analysis cost of pathological examination at the IPM [1]. The co-operation between the laboratory of the IPM and the two French laboratories in the realization of the examinations which purely were free helped us to have these results. For the other malagasy laboratories, the cost accessibility of the population to have these examinations and the lack of sufficient infrastructure for their realization would be the major obstacles to practice them in routine. We will talk about a small population found at the IPM, we have to remember that it was an almost exhaustive study.

The hormonal profile of a population may change over time as it has been reported in the United States by Li et al. between 1992 and 1998. Indeed, the rate of ER+ and PR+ respectively rose from 75.4 % to 77.5 % and 65.0 % to 67.7 % between the two periods. The authors then discussed the role of hormonal factors [3].

According to Huang, several risk factors are accompanied by an increased risk of breast cancer with ER+ and PR+ [4]. These situations are hormonal exposure: early menarche, nulliparity, late primiparity, high body mass index, android obesity and possibly the use of oral contraceptives. The risk of having breast cancer with ER+ and PR+ is higher in postmenopausal women. According to Rafaramino the percentage of nulliparous women is only 13 % and 60 % of women in her series were not yet at menopause [2]. In our study, we could not identify menopausal status of our population study. But we remarked that the rate of HR+ breast cancer increases with age.

In contrast, the risk of breast cancer ER- and PR- increases if there is a family history of breast or ovarian cancer in the first degree, also there is no role of hormone in these cases [4]. Our study which retrospectively has been realized in a laboratory, did not allow us to collect these information. An exploratory study with collection of data concerning hormonal exposure could help us to affirm or not the existence of these connections among Malagasy women.

ER+ is more frequent among women that live in developed countries than those who live in developing countries [5, 6]. Dey in Egypt talked about differences in HR expression between breast cancer in rural and urban localities. His study indicated that urban women have about 2 to 4 times risk of ER+ incidence rate compared to rural women [7]. This finding corroborates other studies talking about higher exposure to xenoestrogens in urban areas which is related to ER+ cancer [8].

Malagasy low socioeconomic level could explain the fact that there were fewer ER+/PR+ amongst women in our population. During our study period, according to the Malagasy National Institute of Statistics, the rate of Madagascar's real economic growth was negative (- 4.6 %) [9]. The rural population decline for various reasons; from 1975 to 1993 it decreased from 84 % to 77 % of the Malagasy population. Despite this tendency to decrease, the rate of rural population is more important than the urban population [10]. This could be one of explanations of this lower positivity of HR and the higher negativity of them in the analysis of all population.

Our study highlighted a rate of 43 % of profile ER+/PR+ against 52 % in McGuire's series and 53 % in the Carolina Breast Cancer Study [11, 4]. Profile of HR in breast cancer differs from race and ethnicity. That has been observed in the United States between different types of populations. It was demonstrated while Hawaiian and Caucasian non-Hispanic women have higher HR+ (respectively 80.7 % ER+ / 72.2 % PR+and 81.2 % ER+/ 69.5 % PR+) than African American and Korean women (respectively 66.3 % ER+/ 55.8 % PR+and 60.7 % ER+ / 51.2 % PR+) [12]. In comparison with our results, knowing that their study was conducted in patients over 50 years from different ethnic groups, we remarked herein that Malagasy women in this study have their specificity. The status ER+/PR- represented 20.6 % of 50 years of age and older. Compared to all ethnic groups in Li's study in America there was no group which has proportion more than ours; the higher rate was South/central American women with 16.1 % for this HR status [12]. For all the population, we roughly have the same percentage of women with a profile ER+/PR- compared to McGuire results, while this rate is 17 % [11]. The Carolina Breast Cancer Study found 11 % of this type [4]. Our percentage was 16.8 % for this HR status type. More cancer ER+ status among post-menopausal patients is explained by the fact that the progeniture stem cells ER+ are more numerous at that time. In fact, this type of ER is less resistant to mutations and they are more important later in life, so they have time to accumulate mutations according to multi-hit theory of carcinogenesis [13]. In the other hand, ER- stem cells are few in number and resistant to mutations, explaining that they are less important than ER+ breast cancer among patients exposed to estrogen risk factors early in their life [13, 7].

For patients with ER-/PR-, Korean women living in America have the more important percentage with 36.1 %. In practice, for this patients group, there is no indication of hormone therapy. Particularly in the adjuvant setting, it has been recognized for a long time that there is no need to prescribe hormone therapy in patients who do not have HR+ [14]. According to Mc Guire in 1977, there were no patients with this hormonal profile in the metastatic setting, which responded to hormone therapy [11]. For Malagasy women with breast cancer, hormone therapy was prescribed to patients without determination of HR status [2]. In fact, except for patients less than 35 years of age, in whom this type of HR status represented 85.7 %, there will be advantageous to prescribe hormone therapy for some 65 % of the patients. The non-prescription of hormone therapy may leave the two-thirds of the population suffering from breast cancer without treatment. It explains that hormone therapy empirically is prescribed in Malagasy women with breast cancer. But toward our study we suggest determination of the HRS for all patients with invasive breast cancer for more precision in the treatment.

Regarding hormonal profiles ER-/PR+, in our study, the rate of patient was very low; we saw only 2 of 77 patients nearly 3 % with this hormonal profile. This profile type represented 6 % of the study population in the McGuire's series and 8 % for the Carolina Breast Cancer Study [11, 4]. In fact, the chance of finding a PR+ is very low in patients with ER-; this is because genes coding for the PR are under the control of ER [15]. Nadji et al. observed no patients with PR-/ER+ profile in his series [16]. Similarly, in the study of Li, the percentage of patients with a profile ER-/PR+ varies from 2.69 % in African-American to 4.7 % among Koreans [12]. For Olivotto, in a study from 2001 to 2002 on 192 ER- patients, he found 191 (99.5 %) patients with PR-. Also, he questioned the practical use of the dosage of PR because it does not affect therapy [17]. For Malagasy women with breast cancer, if it can affect the cost of the immunohistochemistry determination, ER only would be determined at first time. PR status will be determined in ER- tumor only.

About the proportion of HR+, the rate of ER+ is more important than the rate of PR+. Our series has a proportion of HR+ rate considerably lower than in other studies. Indeed, the rate of ER+ was 60.87 % against 75 % for Nadji, 69 % for McGuire and 64 % for the Carolina Breast Cancer Study. About PR+ rate, the difference is more pronounced. In our case, this rate was 46 % against 55 %, 58 % and 61 % respectively in the series of Nadji, McGuire and the Carolina Breast Cancer Study [16, 11, 4]. By dividing our population into three age groups, we remarked that this proportion increases by age, without statistic differences. For patients less than 35 years of age ER+ was 14.3 % and PR+ 14.3 %; ER+ was 61.1 % and PR+ 47.2 % for patients between 35-50 years which was yet under those found in the literature series [16, 11, 4]. It was different for those which are more than 50 years; ER+ represented 70.7 % and PR+ 52.9 %. The proportion of ER+ was stackable to Native American and South/Central American respectively 71.2 % and 70.4 % [12]. The PR+ proportion was a little more important compared to Korean women (51.2 %) and lower than African American (55.8 %) [12].

## Conclusion

These findings suggest that important studies should be conducted among Malagasy women with invasive breast cancer with consideration of their age. Here, hormone exposure and post-menopausal status of these patients could explain the high rate of HR+ among women more than 50 years [4].
